# Sports practice in Japan and France: A comparative analysis

**DOI:** 10.1371/journal.pone.0253435

**Published:** 2021-06-30

**Authors:** Brice Lefevre, Hiroatsu Nohara, Olivier Nier

**Affiliations:** 1 L-VIS, Univ Lyon—Claude Bernard Lyon 1, Lyon, Rhône-Alpes, France; 2 Aix-Marseille Université, LEST-CNRS, Aix-en-Provence, France; 3 Yamanashi Gakuin University, Kofu, Yamanashi, Japan; Universidade Federal de Juiz de Fora, BRAZIL

## Abstract

This article aimed to compare the sports participation of populations from two distinct societies with huge cultural differences: France and Japan. At a macro-statistical level and using a societal approach based on two national ad-hoc surveys–in contrast with functionalist and culturalist analyses–it analysed the differences in sports participation between the two populations. The angle adopted for analysing sports participation was that of voraciousness (number and types of sports and physical activities practised). We performed a secondary analysis of a Japanese national ad-hoc survey and a French national ad-hoc survey (quotas sampling, June/July 2016), which reported activities precisely over the preceding 12 months. The two reconstructed samples for comparison concerned people aged between 18 and 70, and 46 PSAs and PSA families, making it possible to evaluate sports participation. The Japanese sample consisted of 2,612 individuals and the French sample of 3,791 individuals. To identify statistically significant differences between the two samples, Fisher’s exact test and bootstrap approaches was used (boot package in R software). Sports participation and voraciousness for sport are higher in the French population than in the Japanese one. For the overall rate of sports practice, there is a difference of 8.2 points (95%CI[6.0;10.3], p<0.001), and the difference concerning the average number of activities practised by practitioners is 1.7 activity (95%CI[1.9;1.5], p<0.001). In both countries, young males have the highest level of sportiness. Age is an important variable with a lower difference between the youngest and oldest age groups for sports participation in Japan than in France: when compared to France, the difference in difference for the rate of no activity is 13.7 points (95%CI[6.3;21.1], p<0.001) and -2.3 activities for the average number of activities among practitioners (95%CI[-3.0;-1.7], p<0.001). Some physical and sports activities (PSAs) are specific to France (e.g. skiing with 12.7 points difference, 95%CI[11.3;14.1], p<0.001) and others to Japan, such as baseball (with 9.8 points difference 95%CI[8.6;11.0], p<0.001) or more traditional PSAs like Japanese calisthenics and radio exercise (at 15.9%). In contrast to France, Japan is still in the process of greatly modernising its sporting tradition as a result of its particular cultural dimensions. We can identify physical and sports activities which are specific to each country, as well as similar activities in the two countries and wider diversification in France. Voraciousness for sport is higher in France than in Japan. In both countries, the youngest age groups and men have the highest level of sportiness. Yet, the difference between the youngest and the oldest group is smaller in Japan due to the pressure of work.

## Introduction

The aim of this study was to investigate the practice of sports among two very different populations. To our knowledge, no scientific articles on the recent ‘sportiness’ of the Japanese and French populations have been published in an international journal. To this end, the study compared the appetite for physical and sports activities (PSAs) in Japan and France (number and type of activities practised).

For a better understanding of participation in social activities, one of the best methods is to vary the contexts in which they are observed ([1], Durkheim). The primary interest of this study is both methodological and epistemological. Japan and France are a priori similar in terms of level of economic and social development, but very distant in their cultural, political and institutional history. For instance, in the World Values Survey [2–4], the Japanese and French differ greatly from each other in collective spirit, individualism, hedonism and adventure. In the Organisation of Economic Cooperation and Development (OECD, [5]), there are again huge differences between both countries in average annual working hours and women’s activities in the workplace [6]. Moreover, France is a relevant reference for comparison: it is close to the United Kingdom (Kokolakakis et al., 2012, [7]) which is known to be the inventor of modern sport, and it is strongly influenced by a liking for recreation and ’fun’, such as Californian lifestyle sports, and the consumerist trend [8]. Furthermore, comparable ad-hoc surveys, based on national representative samples, are available for each country. This provides a rare occasion to conduct a relatively reliable comparison. Scientific publications comparing Japan and other countries are rare, even more so with comparative analyses based on empirical data. One of the few relevant examples is a Franco-Japanese comparison of the relationship between female and male activities (work, family and society) [9, 10], which shows the interest of societal approaches (or ‘inter-national’ approaches) to formulate explanatory hypotheses (see also the special issue on Japan in the journal *Sociologie du travail* (Sociology of Work) [11]. Such approaches differ from functionalist or culturalist analyses [12, 13] and aim to overcome the universalist trap of functionalist (or ‘cross-national’) approaches. In the context of non-Western comparisons, particularly with Japan, some historians [14] and sociologists ([15, 16]) stress that functionalist analyses often suffer from ethnocentrism by Westerners projecting their representations on the non-Western nation.

The first challenge is to make data from different cultural universes comparable while avoiding the risk of universalism arising from the pitfalls of semantic equivalence [17]. Nonetheless, cultural differences remain crucial as they reinforce the usefulness of the comparison since comparative analyses are generally made between countries with elements of a common history that make them relatively close culturally and economically. One of the elements of our topic, modern sport, is a recent Western invention, whose particularity is its competitive dimension, that has successfully permeated into the daily lives of people around the world. As a result, many international indicators have been developed to measure people’s well-being, with involvement in PSAs as an important factor. Our aims are to differentiate the ‘universal’ from the ‘specific’ in sports practice and to reveal the complex relationships between society and sport. These aims cannot be achieved by the functionalist approaches with the unidimensional quantitative indicators often used by international organisations. The demands of formalism or the generalisation of a theory often have distorting effects. For instance, in the field of PSAs, surveys conducted by the European Commission, such as Barometer for Sport [18–22] or OECD, suffer from such distortion by seeking to “highlight differences in ‘scores’ from one country to another on aspects and indicators whose continuity is assumed” [12]. Academic studies have followed this kind of approach [23–31] and sometimes criticised it. The crux of the matter is that sport and its definitions are socially constructed objects whose meaning may differ from one country to another [27]. In contrast to this approach, the semi-inductive nature of the societal approach leads to the construction of a specific model for each comparison. The approach aims to gain initial insight into the subject of study while also drawing out the first explanatory hypotheses that must subsequently be tested for a full understanding of the differences and similarities between the observed cases.

To apply a societal approach, we needed two comparable national ad-hoc surveys, and to perform a practical deconstruction of the issue of sportiness. This study approached the sports practice of the population (i.e. the actual practice of PSAs by an individual) from the angle of sports voraciousness. Derived from the sociology of culture and developed by Katz-Gerro and Sullivan [32], the concept of voraciousness is a quantitative reflection of leisure consumption: the number of sports and physical activities practised in our case. In this respect, it is an “important discriminator in social analysis” [32], p. 194 and a useful way to measure sports engagement. Voraciousness is thus defined not just as engaging in many activities, but also as a “commitment not to leave many activities untouched or unpractised” [33]. A sports voraciousness approach can therefore lead to the observation of significant social differences in the sports practices of populations [7]. This approach should enable us to deconstruct sportiness by taking into account all activities declared in surveys and thus avoid the pitfalls resulting from an overly functionalist approach using, for example, questions based on subjective declarations of whether or not physical and sports activities in general are practised [34]. What is more, breaking down sports practice by activity makes it possible to analyse the weight of the PSAs identified and gain greater demographic insight into the country’s sports landscape. Finally, sports voraciousness is a quantitative continuum, allowing a more nuanced and progressive understanding characteristic of the social sciences. Yet while quantitative surveys are indispensable, they do not eliminate the need to consider the contexts and the connections between the actors and these contexts.

Taking a step back, while it is undeniably essential to analyse the responses to surveys, this data also needs to be combined with macroeconomic data and knowledge on the organisation of society and lifestyles, navigating between micro-, meso- and macro-level analyses, even if the data concerns individuals out of context. This requires in-depth knowledge of the relationships between the topic of study and each of the two societies, i.e. of both the data produced and the context of its production. These elements were taken into consideration in our interpretation of the results, which highlight salient points that potentially explain the behaviour observed. This study can be seen as a first step in a quantitative societal analysis which, we posit, has much to contribute to knowledge production, in contrast to standard functionalist approaches using a quantitative method. The Methods section presents the tools used to deconstruct sportiness, compare voraciousness in Japan and France, and also compare the weight of the PSAs.

## Materials and methods

### The selection of two comparable surveys

In quantitative sociology and for an international comparison with a societal approach, the primary collection data is very expensive and is very complex. That’s why our analysis of data was a secondary analysis: the surveys have originally been designed by different researchers and for different purposes [35]. Difficulties in understanding Japanese surveys and differences in language and culture [36] created problems in finding data concerning the relationship between the Japanese population and PSAs, as well as in translating and interpreting the questionnaire and the questionnaire database. Two English-language sources guided the research: a Nippon foundation’s sports-life survey [37] and Wolfram Manzenreiter’s book Sport and Body Politics in Japan [38].

An initial comparison was then made using two national surveys, one Japanese and one French, carried out in the summer of 2016 (Sport life survey 2016, Baromètre National Sports de nature 2016). They served as a source for the investigation. Both are the result of a series of national surveys conducted over a period of at least 25 years. In the Japanese survey [39], data was collected from people aged between 18 and over, living in Japan and taking part in sports and physical activities. All data were fully anonymized before we accessed them. Questions focused on the frequency, duration, intensity and range of the activities, including spectating and sports volunteering. The data was gathered via an official home visit survey, either by handing over the paper questionnaire and retrieving it a week later or face-to-face if the questionnaire was not self-administered. In total, data was collected on 3,000 individuals. There were 300 locations (273 cities and 27 towns/villages) and the extracting method was by quota (based on gender, age and region). The survey period was June 10th to July 10th, 2016. The survey crude sample characteristics are in Table 1.

**Table 1:** Japanese national survey and French national survey crude characteristics.

Characteristics	Japan	France
	n (%)	n (%)
Sex (male)	1491 (49.7)	1933 (48.2)
Age (15–19 years old)	_	283 (7.1)
Age (18–19)	74 (2.5)	_
Age (20–29)	393 (13.1)	664 (16.5)
Age (30–39)	499 (16.6)	721 (18.0)
Age (40–49)	570 (19.0)	743 (18.5)
Age (50–59)	474 (15.8)	663 (16.5)
Age (60–69)	557 (18.6)	940 (23.4)
Age (70 and more)	433 (14.4)	_
Overall	3000 (100)	4014 (100)

Source: ‘Sports-Life Survey’, Nippon Foundation, 2016 and ‘Baromètre national des sports de nature’, Ministry of sports, Union Sport & cycles, Lyon 1, 2016.

In the French survey [40], data was collected via an online, self-administered questionnaire from people living in France and aged between 15 and 70. All data were fully anonymized before we accessed them. Questions focused on sport and physical activity participation (and especially outdoor sports), motivations and sport-related expenditures. In total, data was collected on 4,014 individuals selected by quota. A final reweighting was performed based on gender per age, household size, diploma, region and town size. The survey period was near that of the Japanese survey: July 4th to July 18th, 2016. The survey crude sample characteristics are in Table 1.

A methodological study [41] shows that the main problem is not the season or the duration of the surveys’ fields or the season sports practise but the subjective definition of the PSAs. The method of questioning concerning PSA practice was similar in both surveys, with the reference period the preceding 12 months and a list of activities to be indicated with yes or no. The two selected surveys were also unique in questioning people in detail about all their PSA practices. In all, 85 activities were included in the French survey and 125 activities in the Japanese survey. We can thus consider that the main PSAs practised are present, making it possible to investigate the sports practice of the two populations through an analysis of the declared activities. In both surveys, the definitions of PSA practice are based on a very broad understanding of the activity. Lastly, in order to fully understand the social processes behind the production of the quantitative data for each survey, both the Japanese and the French survey managers were interviewed.

### Data alignment for comparability

In order for the comparison to be viable, it was first necessary to standardize the fields of the two surveys, i.e. to ensure consistent field strength [42]. As the scope of the Japanese survey was ages 18 and more and that of the French survey ages 15 to 70, we used the largest overlapping range: ages 18 to 70. After this adjustment, the Japanese survey went from 3,000 to 2,612 individuals and the French survey from 4,000 to 3,791 individuals (Table 2). With the objective of comparability, four rules concerning the coding and grouping of PSAs were defined. First, activities peripheral to PSA were removed (e.g. camping, etc.). The removal of these activities did not impact the overall results. Second, only activities that were identical in both surveys were retained. Third, when some categories of activities were subdivided in one survey, these were regrouped to be identical with the other survey. Fourth, when an activity was significantly practised (>5%), it was identified as such in the results even if it was part of a broader group of activities.

**Table 2:** Japanese and French national surveys characteristics for comparability.

Characteristics	Japan	France
	n (%)	n (%)
Sex (male)	1316 (50.4)	1857 (49.0)
Age (18–30)	506 (19.4)	910.4 (24.0)
Age (31–40)	508 (19.4)	729.2 (19.2)
Age (41–50)	573 (21.9)	806.2 (21.3)
Age (51–60)	479 (18.3)	675.0 (17.8)
Age (61–70)	546 (20.9)	670.2 (17.7)
Overall	2612 (100)	3791 (100)

Source: ‘Sports-Life Survey’, Nippon Foundation, 2016 and ‘Baromètre national des sports de nature’, Ministry of sports, Union Sport & cycles, Lyon 1, 2016.

### The analysis design

In the first stage of the research, the aim was to build a base for each survey that isolated each PSA and the main socio-demographic variables in order to carry out the quantitative semi-inductive work. In parallel, an analysis of the different definitions of sport in the construction of the two surveys and the structural differences of the questionnaires allowed us to grasp some qualitative aspects of PSA. Quantitative comparisons were then made on the number of activities practised, the index of concentration of activities (with and without the removed PSAs, and with and without groupings), the popularity of activities, and finally the comparison of activities between the two countries. The two socio-demographic variables, age and sex, which were directly comparable and are the major factors explaining PSA, were crossed with the number of practices declared in this first work (Table 3).

**Table 3:** Level of sports voraciousness in France and Japan.

	Japan n	% [95% CI]	France n	% [95%CI])	Difference points [95%CI]	P (Fisher’s exact test)
No activities	709	27.1 [25.5;28.9]	719	19.0 [17.7;20.3]	8.2 [6.0;10.3]	<0.001
1 activity	584	22.4 [20.8;24]	456	12.0 [11;13.1]	10.3 [8.4;12.3]	<0.001
2 activities	491	18.9 [17.3;20.4]	741	19.6 [18.3;20.9]	-0.7 [-2.7;1.2]	0.460
3 activities	318	12.2 [11;13.5]	510	13.4 [12.4;14.6]	-1.3 [-3;0.4]	0.140
4 activities	205	7.8 [6.9;9]	317	8.3 [7.5;9.3]	-0.5 [-1.9;0.9]	0.486
5 activities	129	4.9 [4.2;5.9]	236	6.2 [5.5;7.1]	-1.3 [-2.5;-0.1]	0.032
6 activities	52	2.0 [1.5;2.6]	163	4.3 [3.7;5]	-2.3 [-3.2;-1.4]	<0.001
7 activities	45	1.7 [1.3;2.3]	139	3.7 [3.1;4.3]	-1.9 [-2.7;-1.1]	<0.001
8 activities	32	1.2 [0.9;1.7]	108	2.8 [2.4;3.4]	-1.6 [-2.3;-0.9]	<0.001
9 activities	20	0.8 [0.5;1.2]	82	2.2 [1.7;2.7]	-1.4 [-2.0;-0.8]	<0.001
10 or more activities	27	1.0 [0.7;1.5]	321	8.5 [7.6;9.4]	-7.4 [-8.4;-6.4]	<0.001
Overall	2612	100	3791	100		
Practitioners average Number of activites	1903	2.8 [2.7;2.9]	3072	4.5 [4.3;4.7]	-1.7 [-1.9;-1.5]	<0.001*

Source: ‘Sports-Life Survey’, Nippon Foundation, 2016 and ‘Baromètre national des sports de nature’, Ministry of sports, Union Sport & cycles, Lyon 1, 2016.

* bootstrap significance test.

We carried out a secondary analysis, that is to say that we have yet our two samples and do not try to define a future sampling scheme. The aim is simply to check if the given samples sizes are able to detect differences to avoid a type II error. We performed a statistical power analysis based on power calculations according to Cohen [43] using the pwr and PASSED packages [44, 45] on R software for comparing means with binary or count data. In line with Cohen’s rules of thumb, we used the conventional threshold of 0.8 for the power of test and an alpha level of 0.05 (two-sided). The aim was to compare percentages of binary data (for instance the rate of no activity) and means of count data (the number of PSAs) between the Japanese sample and the French sample. The distribution of the outcome variable for binary data is Bernoulli (pwr.2p2n.test function of pwr package) and the distribution of the outcome for the mean with count data is Poisson (power_Poisson of PASSED package). Since a rule of thumb in social sciences is to try to detect medium effect sizes (0.5). And for the number of PSAs, we tried to detect a difference of one activity. In the case of binary data, the minimal target sample size was 62.8 units for each group, and for means with count data, 72.9 units for each group. The Japanese sample size was 2,612 individuals and the French one 3,791. Since subsets were created according to sex (mainly 50% of the sample for women) and age (mainly 20% of the sample for each age group), we know that the identified thresholds complied.

To check this fact, for each category, the number of individuals is indicated in Tables 3–7.

**Table 4:** Sportiness by age and sex in Japan and France.

Country n	Categories n	Rate of no activity	Average number of activities (at least one PSA declared)
		% [95% CI] (Fisher’s exact test)	[95%CI] (Bootstrap significance test)
	SEX		
Japan (2,612)	Female (1296)	30.4 [27.9;33.0]	2.7 [2.6;2.8]
	Male (1316)	23.9 [21.7;26.4]	3.0 [2.8;3.1]
	Difference female male	6.5 [3.0;9.9] (p<0.001)	-0.3 [-0.5;-0.1] (p = 0.001)
France (3,791)	Female (1,934)	20.4 [18.6;22.2]	4.2 [4.0;4.5]
	Male (1,857)	17.5 [15.8;19.4]	4.8 [4.5;5.1]
	Difference female male	[0.3;5.3] (p = 0.030)	-0.5 [-0.9;-0.1] (p = 0.004)
	AGE		
Japan (2,612)	18–30 (506)	23.3 [19.8;27.3]	3.2 [2.9;3.4]
	31–40 (508)	23.8 [20.2;27.8]	3.2 [2.9;3.4]
	41–50 (573)	26.2 [22.7;30.0]	2.9 [2.8;3.1]
	51–60 (479)	33.6 [29.4;38.1]	2.3 [2.2;2.5]
	61–70 (546)	29.1 [25.4;33.2]	2.5 [2.3;2.6]
	Difference 18–30 61–70	-5.8[-11.3 -0.3] (p = 0.039)	0.7 [0.4;1.0] (p<0.001)
France (3,791)	18–30 (911)	11.1 [9.1;13.3]	6.1 [5.6;6.7]
	31–40 (729)	13.1 [10.8;15.8]	4.9 [4.5;5.2]
	41–50 (806)	20.0 [17.3;22.9]	4.1 [3.8;4.4]
	51–60 (675)	23.3 [20.2;26.7]	3.3 [3.1;3.6]
	61–70 (670)	30.6 [27.1;34.2]	3.1 [2.8;3.3]
	Difference 18–30 61–70	-19.5 [-23.7;-15.3] (p<0.001)	3.0 [2.5;3.6] (p<0.001)

Source: ‘Sports-Life Survey’, Nippon Foundation, 2016 and ‘Baromètre national des sports de nature’, Ministry of sports, Union Sport & cycles, Lyon 1, 2016.

**Table 5:** Differences in sportiness by age and sex between Japan and France.

Categories	Differences in the rate of no activity between Japan and France Diff [95%CI] (p, Fisher’s exact test)	Differences in the average number of activities (at least one PSA declared) between Japan and France [95%CI] (p, Bootstrap significance test)
SEX		
Female	10.0 [6.6;13.5] (p<0.001)	-1.5 [-1.7;-1.3] (p<0.001)
Male	6.4 [3.1;9.8] (p<0.001)	-1.7[ -2.0;-1.4] (p<0.001)
Female-male difference	3.6 [-1.1;8.4] (p = 0.931)*	0.2 [-0.2;0.6] (p = 0.840)
AGE GROUP		
18–30	12.3 [7.4;17.1] (p<0.001)	-3.0 [-3.6;-2.5] (p<0.001)
31–40	10.7 [5.6;15.7] (p<0.001)	-1.8 [-2.2;-1.4] (p<0.001)
41–50	6.2 [0.9;11.4] (p = 0.011)	-1.1 [-1.4;-0.8] (p<0.001)
51–60	10.3 [4.4;16.2] (p<0.001)	-1.0 [-1.3;-0.7] (p<0.001)
61–70	-1.4 [-7.1;4.2] (p = 0.306)	-0.6 [-0.9;-0.3] (p<0.001)
Difference between 18–30 & 61–70	13.7 [6.3;21.1] (p<0.001)*	-2.3 [-3.0;-1.7] (p<0.001)

Source: ‘Sports-Life Survey’, Nippon Foundation, 2016 and ‘Baromètre national des sports de nature’, Ministry of Sport, Union Sport & cycles, Lyon 1, 2016.

* bootstrap significance test.

**Table 6:** PSAs in Japan and PSAs in France by rank.

Country	PSAs (major activity types)	%
**Japan ages 18–70 (n = 2,612)**	Walking	41.9
	Fitness / gym non-competitive exercise (including light calisthenics, radio exercises, etc. 15.9%)	21.4
	Weight training	14.7
	Swimming (including swimming 7.8% and beach bathing 6.9%)	12.8
	Bowling sports (including bowling 10.6%)	10.7
	Running (including jogging and running 9.6%)	10.5
	Golf (including on golf courses 7.0% and driving ranges 6.5%)	10.1
	Baseball (including catchball 6.0%)	9.8
**France ages 18–70 (n = 3,791)**	Hiking (including Nordic walking 6.0%, hiking 35.4%)	42.9
	Cycling (including mountain biking 15.7%)	30.1
	Fitness / gym non-competitive exercise (including fitness 10.5%, indoor cycling 8.3%, aerobics 6.5% and water aerobics 6.0%)	23.4
	Walking	21.9
	Swimming / bathing	21.1
	Skiing (including downhill 11.2%, cross-country 5.6%)	16.2
	Running (including running / jogging 10.8%)	16.1
	Other water sports	14.4
	Football (soccer)	12.3
	Bowling games (including pétanque 10.8%)	10.8
	Other outdoor sports	10.8
	Fishing	10.7
	Ping-pong	10.5
	Tennis	10.0
	Climbing sports (including zipline activities 9.3%)	9.8

Source: ‘Sports-Life Survey’, Nippon Foundation, 2016 and ‘Baromètre national des sports de nature’, Ministry of sports, Union Sport & cycles, Lyon 1, 2016.

**Table 7:** PSAs more practised in Japan than in France and PSAs more practised in France than in Japan.

Population aged 18–70	Rate in Japan (n = 2,612)	Rate in France (n = 3,791)	Difference	p (Fisher’s exact test)
	% [95% CI] (n)	% [95% CI] (n)	Diff [95% CI]	
Walking	41.9 [40.0;43.8] (1095)	21.9 [20.6;23.2] (829)	20.0 [17.7;22.4]	<0.001
Weight training	14.7 [13.4;16.2] (385)	9.3 [8.4;10.3] (354)	5.4 [3.7;7.1]	<0.001
Golf	10.1 [9.0;11.3] (263)	2.5 [2.0;3.0] (94)	7.6 [6.3;8.9]	<0.001
Baseball	9.8 [8.7;11.0] (256)	0.0 [0.0;0.1] (0)	9.8 [8.6;11.0]	<0.001
Ice skating	1.7 [1.3;2.3] (45)	0.7 [0.5;1.0] (26)	1.0 [0.4;1.6]	<0.001
Other team sports	1.7 [1.2;2.3] (44)	0.0 [0.0;0.1] (0)	1.7 [1.2;2.2]	<0.001
Other outdoor sports	0.8 [0.5;1.2] (20)	10.8 [9.8;11.8] (409)	-10 [-11.1;-8.9]	<0.001
Other water sports	0.8 [0.5;1.3] (22)	14.4 [13.3;15.5] (545)	-13.5 [-14.7;-12.3]	<0.001
Skiing	3.5 [2.8;4.3] (91)	16.2 [15.1;17.5] (615)	-12.7 [-14.1;-11.3]	<0.001
Cycling	7.0 [6.1;8.1] (184)	30.1 [28.7;31.6] (1142)	-23.1 [-24.9;-21.3]	<0.001
Hiking	7.3 [6.4;7.4] (191)	42.9 [41.3;44.5] (1625)	-35.6 [-37.5;-33.7]	<0.001

Source: ‘Sports-Life Survey’, Nippon Foundation, 2016 and ‘Baromètre national des sports de nature’, Ministry of sports, Union Sport & cycles, Lyon 1, 2016.

Univariate and bivariate analyses were computed using R 3.5.2 statistical software [46]. Concerning univariate analyses, 95% confidence intervals were performed with bias-corrected and accelerated (“bca”) confidence intervals which are a refined version of the percentile bootstrap (resampling method to mimic the sampling process), using the confintr R package [47] with ci_proportion and ci_mean R functions. For each confidence interval, 50,000 resamples were drawn from the survey dataset with replacement [48].

Bivariate analyses were conducted using bca confidence intervals (50,000 resamples) with ci_mean_diff R function (confintr R package). Concerning the bivariate statistical tests, we computed p-values using Fisher’s exact test to test the differences in proportions (analysis of a 2 × 2 contingency table with the fisher.test R function). We used bootstrap significance tests (50,000 resamples) to test the differences in means because we were unable to use a usual t-test (non-normal distributions) directly [49]. Lastly, bootstrap significance tests (50,000 resamples) were then again performed to compute the p-values of the difference-in-differences of proportions and the difference-in-difference of means. The ways the p-values were computed (Fisher’s exact tests or bootstrap significance tests) are indicated in each table of results. The Scientific Committee of the Sports Policy Institute of Japan and the Sport Scientific Committee of the Ministry of sport of France approved the questionnaires and survey designs. For both surveys, the consents obtained were oral and the data were analysed anonymously.

## Results

We found significant qualitative differences in the themes and PSA list of the questionnaires. The Japanese questionnaire themes were more directed towards health (energy expenditure and health behaviour), as well towards institutional and competitive sport, with parts of the questionnaire concerning the respondents’ past and present club membership, sports events and volunteering. The French questionnaire had no specific parts like those of the Japanese questionnaire and was focused on the analysis of physical and sports activities, considering sport as part of people’s lifestyles. Clubs and competition were only mentioned in the global questionnaire concerning the ways in which a PSA was practised. The Japanese definition was more extensive, involving semi-artistic (such as Japanese drumming) and semi-touristic activities (such as camping), while the French survey focused more on traditional sports (in the English sense of the term) and leisure sports activities. These differences were nevertheless marginal. The Japanese survey identified 120 different PSAs practised and the French survey identified 85. This greater diversity on the Japanese side was notably due to activities aimed at specific populations, such as women (e.g. women-only fitness or maternity exercises) or the elderly (e.g. gateball and many other sports revisited for these participants), as well as to the continued existence of traditional activities (e.g. Japanese calisthenics, Japanese dance or Japanese archery) and variations of activities (e.g. individual baseball training with machines).

Taking into account this diversity of activities made it possible to gain insight into the sports landscapes of the two countries as a whole. The activities were recoded into groups for comparison between the two countries. The activities were then compared to evaluate differences in the popularity of practices, aggregation of practices, and rates. In our comparison base, there were 46 PSAs on the Japanese side and 43 on the French side. This base included all significant activities named in both countries, and these activities defined what constituted physical and sports practice for this comparison.

### The level of sports voraciousness (number of activities practised) in France and Japan

In 2016 (Table 3), the rate of non-participation in sport (i.e. not declaring at least one practice) in the Japanese population aged 18–70 was significantly higher (27.1%) than in the French population (19.0%) (8.2 points difference, p<0.001). This is consistent with a 2007 survey by the International Social Survey Programme [50] which showed a significant difference in the overall rate of PSA practice in Japan and France (14.4 points, p<0.001). Moreover, among practitioners, the average number of PSAs practised by the Japanese was 2.8 activities, while it was 4.5 activities for the French (-1.7 activity difference, p<0.001). Overall, within the scope of the survey, the Japanese were more likely to be single-sport practitioners (including walking and strolling at 39.6%) than the French. While 22.4% of the Japanese practised one activity, this was the case for only 12% of the French (10.3 points difference, p<0.001). Looking at the multi-practice of sports, this was more prevalent among the French. However, in the category of 2 to 5 activities practised, the difference between the two populations was not very significant (reaching a maximum of 1.3 times more for French survey responders). From 6 or more activities, this difference between the populations became more pronounced (a ratio of 2 or, for 10 or more activities, a ratio of almost 8, p<0.001). These results indicate that among practitioners in Japan, slightly less than one person in ten (9.2%) has a high level of sports voraciousness (6 or more activities), whereas in France, this figure is higher than one in four practitioners (26.4%). In terms of the diversification of sports practices, when calculating the number of different activities that make it possible to obtain more than 80% of the total declared practices, only 13 PSAs are needed for Japan, while 19 are needed for France.

In first socio-demographic analysis (Tables 4 and 5), analyzing the difference in sportiness between men and women in Japan, the women’s rate of non-practice (30.4%) was higher than the men’s (23.9%) with 6.5 points difference (p<0.001). In France, this difference in rate between women (20.4%) and men (17.5%) was small (p = 0.030). Among the 18-70-year-olds, the difference in the non-practice rate between men and women in Japan was not greater than the difference in non-practice between men and women in France (p = 0.931). And among practitioners in Japan, the difference in the average number of practices reported for women (2.7 activities) was lower (-0.3 activity, p = 0.001) than for men (3.0 activities). Likewise for practitioners in France, the difference in the average number of practices reported for women (4.2 activities) was lower (-0.5 activity, p = 0.004) than for men (4.8 activities). Among practitioners in the 18–70 age group, there was no difference between Japan and France concerning the difference in the average number of practices for men and women (p = 0.840). Depending on the age, there were differences in the trend observed in the rate of practice and the average number of activities reported. In Japan, there was relative stability compared to France where there was a rapid and almost linear decrease, which is a classical result. And between the 18–30 and 61–70 age groups, there was a difference of -5.8 points (p = 0.039) in the rate of non-practitioners in Japan while, in France, there was a -19.5 points difference (p<0.001) in the same rate. The difference in the rate of non-practitioners between the 18–30 and 61–70 age groups was higher in France than in Japan (13.7 points difference, p<0.001). And between the 18–30 and 61–70 age groups, there was a difference of 0.7 practices among practitioners in Japan (p<0.001) while, in France, there was a difference of 3.0 practices among practitioners (p<0.001). Among practitioners, the difference in the average number of activities between the 18–30 and 61–70 age groups was higher in France (-2.3 activities, p<0.001). While sportsmanship in France was much higher among the youngest (12.3 points differences for non-practice, p<0.001, -3.0 activities, p<0.001), in the last age group, the rates and average numbers of activities were not very different between the two countries (no difference in the rate of non-practice p = 0.306, -0.6 activity [-0.9;-0.3] p<0.001).

### The most popular PSAs in Japan and France

To analyse the differences in the most common activities practised, those with a rate of at least 10% were taken into account.

In Japan (Table 6), walking (including strolling) was clearly predominant, with 41.9% of participants practising this activity. In second place came fitness/non-competitive exercise, which represented one person out of five (21.4%). Weight training stood at 14.7%, swimming at 12.8% and bowling at 10.7% (corresponding to bowling sports overall). Finally, running, golf and baseball accounted for one person in ten.

In France (Table 6), a group of activities was identified as being predominant, with a rate of more than 20% of surveyees practising them: hiking (42.9%), cycling (30.1%), fitness/non-competitive exercise (23.4%), walking (21.9%) and swimming (21.1%). A second group consisting of six PSAs was practised by between 10% and 20%: skiing and running (16.2% and 16.1%), other water sports (14.4%), football (12.3%), then bowling games, other outdoor activities and fishing (at around 11%). Finally, at around 10% or just under, there was table tennis, tennis and ziplining. By comparing the penetration rates of the most important activity types, it is logical that more PSAs are practised in France than in Japan, since sports practice is generally more developed in France. Only six of the 46 activities or comparable groups of activities were practised more in Japan than France (all p-values at <0.001). This was equally observed in the ISSP survey in 2007.

Of these activities (Table 7), the main ones were walking (20.0 points difference, p<0.001), weight training (5.4 points difference, p<0.001), golf (7.6 points difference, p<0.001) and baseball (9.8 points difference, p<0.001). In particular, walking was much more widely practised in Japan (with a difference of 20 points). It is nevertheless important to mitigate this result given the subjective definition of this activity (e.g. it could include walking a dog around the block). This is one of the primary difficulties in the quantitative analysis of sports engagement based on self-declaration, to which is added the risk of semantic equivalence, as words are part of a system of historical meaning that calls for vigilance concerning misleading translations of similar terms with different meanings in questionnaires.

Of the 46 PSAs or activity groups, 33 were more widely distributed in France than in Japan. Of these (Table 7), those with the largest difference (a threshold of more than 10 points difference) were hiking (35.6 points difference, p<0.001), cycling (23.1 points difference, p<0.001), skiing (12.7 points difference, p<0.001), other water sports (13.5 points difference, p<0.001) and other outdoor sports (10.0 points difference, p<0.001). These activities belong more generally to the category of outdoor physical activities or nature sports. In the 2007 ISSP survey [50], although the questions were not the same, hiking and cycling equally came out as the PSAs with the largest difference in practice between the two countries. In our analysis, the activities with the second largest difference (5 points difference threshold, all p-values < 0.05) were swimming, running, football, tennis, table tennis, horse riding, other motorised sports, snorkelling and ziplining.

Finally, five activities were ‘neutral’, i.e. no significant differences (p>0.05) were observed between the two countries. These sports with similar ranks were fitness/non-competitive exercise (p = 0.074), bowling sports (p = 0.870), volleyball (p = 0.130), snowboard (p = 0.223) and other martial arts(p = 0.936).

## Discussion

Before discussing the results, it should be noted that in the empirical sciences, interpretation involves the search for a more or less direct causality between the stated facts. To put it another way, paraphrasing Passeron, it is a matter of reformulating “the meaning of a relationship between descriptive concepts which, in order to transform this meaning […], must involve the comparison of this relationship with empirical descriptions that do not assume exactly the same ‘universe of discourse’ as the relationship interpreted” [51], p. 401). The empirical descriptions that we borrow to interpret our comparative analysis come mainly from the sociology of culture and political sociology, specifically on the countries concerned and on the subject of sport. In this study, the cultural (in particular the relationship to the body), historical, economic and organisational aspects are exploited in a heuristic way to interpret our qualitative results (differences in the sports participation questioning) and statistical results.

As interpreting these results objectively is difficult due to the cultural distance between Japan and France, this called for a unique analytical approach. This involved an asymmetrical interpretation approach to comprehend our study subject and to avoid using Western stereotypes or referring to ‘Japaneseness’ [15]. Current sports practices in France and their development, quite well surveyed, resemble those of the United Kingdom, traditionally considered the birthplace of modern sport, and likewise follow the main trends observed in many other Western countries. So to interpret the differences observed between the two countries, we decided to focus on the Japanese case. Two main works were used; these deal in particular with amateur sports and fitness practices in Japan. The first is Japanese Sports by Guttman and Thompson [14], which provides a socio-historical overview. The second is Sport and Body Politics in Japan, a history by Wolfram Manzenreiter [38], which is more of a socio-political analysis. A number of Japanese documents concerning sports, and social and natural environments were referred to.

Our analysis found that the main PSAs practised in Japan can be grouped by four non-exclusive themes: 1) practices for maintaining good physical and mental health, 2) practices that hone performance/competitiveness/discipline in line with modern sport, 3) collective and/or supervised practices (as opposed to independent, personal practices) and lastly, 4) practices influenced by recreation and ‘fun’, e.g. Californian lifestyle sports and the consumerist trend.

### Fitness and maintenance activities: The norm of physical and mental fitness

In today’s neoliberal context, social norms in contemporary Japan tend to emphasise individual responsibility for maintaining good health in the objective of remaining able to work (even in old age) and of not burdening the nation in terms of health costs. Manzenreiter observed this trend towards the promotion of individual health in the broadest sense and, more specifically, through sport: in his words, the social order “ultimately requires the individual to be responsible for his or her physical condition, health and well-being” [38]. Moreover, the national Japanese survey includes the theme of health behaviours, whereas the national French survey does not. Activities that correspond with this goal–walking (41.9%), fitness & non-competitive exercise (21.4%) and weight training (14.7%)–rank at the top of Japanese practices. It should also be noted that light calisthenics and radio exercises are an important part of this family, still concerning 15.9% of individuals. Last, let us note the important place of bathing/beach bathing (6.9%) of the population studied. In France, this norm is also present–fitness & gym (non-competitive exercise, (23.4%) are as widespread as in Japan–but in a less exclusive way. The categories of walking and weight training are more widespread in Japan than in France (respectively 20.0 points difference, p<0.001 and 5.4 points difference, p<0.001).

### The historical presence of modern sport: Being disciplined (rules) and productive (competition)

We also observe a significant development of body practices in Japan that conform to the goals of modern sport and are part of “the production society with its emphasis on achievement and success” [38]. In Japan, sport has historically been a state tool supporting nation-building and enabling a form of social control, above all with respect to hierarchy. Even today, sport is considered a way to improve productivity (including optimisation and performance) and to reinforce mind and spirit, health, morality, solidarity, team loyalty, national identity and quality of life and health. This aspect is especially notable in the schooling period for children and young adults [52]. Young people are encouraged to take part in daily sports and physical activities, both within and outside the school curriculum, mainly in a competitive format. The most practised activities are the traditional sports practices in the English meaning of the term (regulated, institutionalized, competitive sports). Furthermore, in the national Japanese survey, the themes of clubs, competition and sports events are developed. This is not the case for the national French survey. Since the Meiji Era (1868–1912), the global cultural phenomenon of sport has also penetrated Japan, with local practices (e.g. traditional martial arts and games) supplemented by ‘modern’ sports activities. The dominant social classes of the population understood that they could take advantage of the “Western imperatives of autonomy and control of the body” [38] for themselves and for the nation, both internally and externally, with a concern for Japan’s place in the international arena. The impact of Western culture on Japanese society resulted in the importation of modern sport and a modification of the local body culture. Thus, we find typical modern sports among the most practised activities such as bowling sports (especially ten-pin bowling, 10.6%), golf (a sport metaphor for workplace hierarchy, 10.1%) and baseball (9.8%). Baseball is a sort of ‘ideal’ sport that Thompson often uses as a reference to explain the modernization of the Japanese PSAs landscape. In his words: “baseball and its collective virtues–teamwork, self-discipline, self-sacrifice–(…) in middle and high school, baseball continues to be played more widely than football (at least among boys)” [53]. In this respect, baseball is a symbol of asceticism and characteristic of modern sport in its regulatory, collective and competitive characteristics, as well as in the strict discipline of the supporters’ clubs (baseball, also highly professionalized, can be the way to earn a great amount of money when a player is selected for the professional team, in the same way as football in France). However, statistically catchball (6.0%) represents the majority of practitioners of this category of sports, raising the question of the development of the practices in Japan and how this should be interpreted. This reinforces the idea that global sports (represented here by the American version of the sport) need to be rooted in historical territorial structures in order to spread [54]; that is, a sport is constantly nourished by local sports culture while in turn transforming it. In this way, the local and the global are not mutually exclusive, but in dialogue, leading to an increase in diversity among populations and even celebrating local culture on occasion [55, 56]. In Japan, of the ‘modern sports’ practised, baseball counts proportionately fewer adult players than other sports, as this sport is above all a spectacle sport, similar to football in Europe. However, golf and baseball, activities that appeared during the Meiji Era, are among the PSAs most specific to the Japanese population and much more present than in France (respectively 7.6 points difference, p<0.001 and 9.8 points difference, p<0.001). While in France, competitive, regulated sports are certainly present demographically and are symbolically important, non-competitive and non-institutionalized activities are largely in the majority. The two most widespread activities in France are hiking (42.9%, 35.6 points difference, p<0.001) and cycling (30.1%, 23.1 points difference, p<0.001), and are strong markers of the French tendency to practise informally, as was also noted in the earlier 1985 French national survey.

### Activities that are institutionalized, supervised and collective

The omnipresence of modern sport in Japan results in the significant practice of institutionalized and supervised PSAs. The social control exercised by the state through sports has probably slowed the development of more individualistic and/or hedonistic PSAs in Japan. As Manzenreiter explains, since the Meiji Era, institutions have worked to ensure that sport is “confined within the social framework of large organisational units”. The introduction of international sports has thus had the effect of modifying traditional local folk games as well as ritualized bodily practices. For example, martial arts have undergone ‘sportification’. The state plays a major role in this process, in particular by introducing a sport into the national education system. Sports have in this way become a tool for training the modern Japanese citizen, a citizen who ‘plays’ collectively. In the World Values Survey [2–4], the Japanese results indicate a people who are collective and trusting. A significant number of respondents (36.6%) state that most people can be trusted, compared to 18.7% for the French (p<0.001). Similarly, the Japanese show greater confidence in the judicial system (77% versus 39.9%, (p<0.001)) and a strong sense of collective responsibility (rating as ‘never justifiable’: Claiming government benefits to which you are not entitled 61.3% versus 41.3%, (p<0.001) / Avoiding a fare on public transport 75.7% versus 49.9%, (p<0.001) / Cheating on taxes 81.3% versus 47.8%, (p<0.001)). Through compulsory programmes, school is the main disseminator of sport in its collective and competitive form. In the words of a Franco-Japanese researcher who has had personal experience of dual French and Japanese schooling: “The Japanese system manages to transform an individual sport into a team spectator sport”. This has had a major influence on the Japanese population’s sporting habits. Until recently, institutionalized and supervised sports did not end with school, but continued throughout life. For employees, workplace sports are encouraged outside working hours, and for older people, events and activities are supervised by local neighbourhood associations (chōnaikai or jichikai, informal/formal community citizens’ committees) [57]. These fall into the category of what Manzenreiter calls corporatist policies (e.g. light calisthenics and radio exercises at 15.9%). Spectator events are also an important element in prioritising the collective over the individual [58] often advocating group solidarity. These elements are present in the questionnaire of the Japanese national survey which asks many more questions on the institutional and collective aspects of physical and sports practice than the French national survey. The themes of clubs, volunteering and sports events are developed in the Japanese questions, which is not the case for the French national survey. In contrast, sports practice in France is more dominated by personal, unsupervised activities, which is indicative of a greater tendency towards individualism. This does not negate the strong presence of collective and institutionalised activities in France, but puts it into perspective.

### Activities that are hedonistic and individualistic: The “fun” wave

While the societal frameworks discussed so far are still very much in evidence, Manzenreiter and Thompson both point to a current trend of decreasing social control by the state. These researchers see this social movement as mainly driven by the younger generations, arguing that with “With the ongoing integration and consolidation of sport, leisure, recreation, television, film and tourism into elements of the entertainment industry, the boundaries between sport and entertainment are dissolving” [38]. In the West, these transformations in the sports landscape have already taken place: we see a hybridization of PSAs through their ‘sportification’ or ‘desportification’, a blurring of boundaries and intensive consumption. More broadly, these transformations are likely to lead in Japan, as they have in Western countries including France, to a mass dissemination and diversification of PSAs. Manzenreiter points out that so-called alternative or counter-cultural sports from the "fun wave" (or ‘fun’ lifestyle sports) typical of the urban consumer in France are also developing in Japan. However, there seems to be a time lag in Japan in the development of these often autonomous sports that stand out in contrast to collective, regulated sports. The WVS survey highlights a lower tendency of individualism and hedonism among the Japanese than the French (Often thinking about the meaning and purpose of life 23.6% versus 44%, (p<0.001) / Agree strongly with ‘I seek to be myself rather than to follow others’ 14.8% versus 72.7%, (p<0.001) / ‘It is important to me to have a good time’; to ‘spoil’ myself is ‘very much’ to ‘somewhat’ like me 18.8% versus 72.6%, (p<0.001)). These results could be explained by the hypothesis of a difficulty in Japanese culture (1) to deal with the insecurity generated by uncertainty, (2) to take individual decisions ‘in action’ (particularly in the framework of nature activities), both resulting from a particular relationship to nature based on mistrust given its sometimes aggressive character in the archipelago [59]. In the WVS results, the Japanese were thus less likely than the French to be concerned by the statement ‘Adventure, taking risks, having an exciting life are ‘very much’ to ‘a little’ like me’ (25.8% compared to 51.8%, (p<0.001)). The overrepresentation of outdoor sports in France is moreover very clear (hiking, snorkelling, other outdoors sport, all p-values at p<0.001).

At the beginning of the 2000s, Thompson observed that ‘California’ lifestyle sports were not as developed in Japan as in countries like the United States. Fitness, which is often practised in urban areas, is the exception: “Southern California is perceived–and not just by young Japanese–as the holy land from which the hedonistic cult of physical fitness has been disseminated”. In Japan, weight training is practised by 15% of respondents, and part of the non-competitive exercise category is specifically concerned with fitness. This hedonistic counter-cultural current is in stark contrast to the asceticism of traditional Japanese or regulated competitive sports: “Conspicuous among those enamoured of surfing and gliding is a turn away from the austere asceticism characteristic of martial arts and samurai baseball” [14]. One can posit that a high level of institutionalization has a limiting influence on the scale of adoption of ‘lifestyle’ activities (fun activities) and the development of practices outside the institutional or collective framework, especially in the context of recreational practices in nature. While ‘fun’, individualistic, hedonistic activities without a collective and institutional framework and/or in nature are practised in Japan, they are not as popular as in Western countries, including France. Though there is a qualitative diversity of activities in Japan, there is less quantitative diversity. Nonetheless, the development of an individualistic, hedonistic counter-culture is correlated to the rise of consumerism and reflects a challenge by the new generation to the strict social rules and conventions of Japanese society. This trend in the adoption of new activities is reflected in the great diversity of practices declared by the respondents (120 PSA identified against 85 in the French survey).

### Explanations for the differences in sports practice (global and portfolio size) between Japan and France

As Manzenreiter points out, “the ethic of diligence and hard work” in Japan implies reduced free time–and therefore less time for leisure sports–thus affecting the overall rate of sports practice (8.2 points difference, p<0.001) as well as the number of activities practised compared to the French (among practitioners -1.7 average number of activities difference, p<0.001). Japanese workers, including executives, take few vacations and have little free time. In France, annual leave is on average five weeks, whereas in Japan, while employees nominally have 21 days of paid leave, they take on average only 6 to 10 days per year. According to the Organisation of Economic Cooperation and Development (OECD, [5]), in 2015, the average annual working time of Japanese workers was 1719 hours per year, whereas for French workers it was 1482 hours. These figures include about 30% of part-time workers in Japan and 18% in France. Concerning only employees with permanent full-time contracts, they work more than 2,000 hours per year in Japan (2024 hours in 2015 [60]). In addition to long hours in the workplace, the lack of long-term leave reduces the time available for leisure in general, including sport.

In our first socio-demographic analysis, we noted that the difference in sportiness between men and women in Japan is not higher than the one observed in France. There is no significant difference between Japan and France in the rate of no activity (p = 0.931) between women and men. Also, between Japan and France, there is no significant difference in difference concerning the average number of activities between women and men (p = 0.840). These results go against what is commonly expected, with larger inequalities between men and women in Japan than in France [55]. According to Dupray and Nohara [6], the overall rate of women in the workplace in Japan is slightly higher than that in France. However, Japanese women’s professional life fluctuates according to their personal life history: they tend to work full-time until the birth of the first child, at which time they withdraw from the labour market. Later they re-enter the labour market, but with less permanent forms of employment (part-time, fixed-term, temporary, etc.). We also noted women-specific activities in the activities reported in the Japanese sample (e.g. women-only fitness or maternity exercises), which was not the case for the French sample. This argument supports the existence of autonomous female practices present in the Japanese PSA landscape, perhaps with forced time availability due to the less permanent forms of employment.

In terms of age, the relative stability of Japanese results is surprising, compared to other countries where advancing age leads to a decrease in the practice. We can see that the youngest are not much more active than the eldest (-5.8 points difference for the rate of no activity, p = 0.039). Also, the average number of activities among practitioners is not much higher (0.7 activity difference for the average number of activities among practitioners, p<0.001). Compared to France, the difference in difference for the rate of no activity is 13.7 points (p<0.001) and -2.3 activities for the average number of activities among practitioners (p<0.001). We can hypothesize that in Japan young people are partly under-practicing because hedonistic and individualistic activities, often counter-cultural and recruiting among young people, are poorly developed. In addition, being a new worker who learns a profession intensively and who will influence the rest of his career. This difference in Japanese youth and French youth may be the result of two different conceptions of life and career. As the working life for the young is more intense in Japan than France, the Japanese youth has lower time availability than the French youth. It can also be hypothesized that the Japanese are the most concerned with their maintenance of good health and more encouraged by the state through physical activity and sport than the French. The time investment in work and correlated lack of leisure time is especially the case for managers, who are generally older in Japan than in France. Seniority in Japan is more highly valued in professional circles and in society as a whole [61]. One of the fundamental differences in the employment system between France and Japan is that there is real social promotion in Japanese companies, which generates trust in social competition. The link to sports competition can be imagined. Conversely, the French might have less confidence in the promotion system than the Japanese.

Another factor to take into account in the interpretation of the results is the Japanese territory itself. Japan is a mountainous island chain with a “violent nature” that means only a little more than one-fifth of the territory can be inhabited [59]. This has led to significant urban concentration. Geographers specializing in Japan even refer to a Japanese Megalopolis [62] made up of three megacities: Greater Tokyo, Greater Nagoya and Greater Osaka. Greater Tokyo, which covers 2% of the territory, is inhabited by one-quarter of the country’s population. This concentration makes space a scarce resource and may help to explain the lower practice of certain PSAs, particularly those in nature (e.g. cycling, hiking, skiing, all p-values at p<0.001). Similarly, indoor and instrumented activities seem to be favoured by the environmental and cultural context. One exception is golf, but this activity is more practised by the upper social categories of the Japanese population, and driving ranges are less space-consuming than golf courses.

Lastly, certain religious values may have an influence on the development of sports leisure activities, reflecting notions of tradition versus modernity, of perceptions of the group and of work [63]. If sports leisure is considered as time for oneself [64], it may come into conflict with traditional and religious values and notions of the primacy of the group over the individual. Individualism and hedonism, which are major drivers of the mass dissemination and diversification of PSAs in the West, do not seem to be so prevalent in Japan. This may be why lifestyle activities that are more self-organised, individual and informal do not seem to have affected Japanese society to the same extent as in France or other Western countries. However, it is interesting to note the relationship to the body in Japan puts high value on an aesthetic and well-maintained body. This is reflected in the fact that fitness activities are among the most popular PSAs (Fitness/gym (non-competitive exercises) 21.4%, weight training 14.7%). While this emphasis on the body can be for personal gratification, it is above all in Japanese culture for others, especially in urban settings.

## Conclusions

Overall, our analysis found that compared to the French population (ages 18 to 70), fewer Japanese practise sports, and those that do practise less types of PSAs, indicating lower sports voraciousness in Japan. There was less diversification (thus greater concentration) in PSAs practised in Japan, mainly in terms of nature recreation. And self-organised practices were also less common in Japan, the norm being rather supervised and/or organised activities. These results are in contradiction with a comparative analysis made between Japan and European Union countries based on a Eurobarometer sport survey [37], a comparison which showed the rate of non-practitioners in France to be 10 points higher than the rate of non-practitioners in Japan. While the time period is different, these findings are in direct contrast to our results. To get a better understanding of the reality, investigating sports practice based on precise PSAs allows more accurate insight into the contrasts between Japan and France, which is not possible with a more general and subjective approach, raising the question of the robustness of the Eurobarometer sport comparison. In our findings, it should be noted that some of the comparative results can run counter to common sense: for example, the popularity of martial arts in Japan is surprisingly no higher than in France–a finding that indicates the influence of Japan on the West, notably through judo, “the Frankenstein’s monster of Japanese sport” [53]. Similarly, two activities established only at the end of the 19th century in Japan, golf and baseball, are much more practised than in France. As Guttman and Thompson argue, there has been a modernization of the Japanese sporting tradition. Yet the inclusion of Western sports and the "fun" wave has not produced an exact replica of what has developed in the West [65] in this country with such a strong culture and traditions [61]. Indeed, these cultural traditions have resulted in a local interpretation of international sports practice. The relationship to the body is both similar and dissimilar, with fewer vernacular activities, less leisure practices typical of the fun wave (individualism/hedonism) and smaller portfolios of PSAs practised. Consequently, and finally, the overall rate of practice that is somewhat lower. This observation is also due to the fact that the rates of practice for the youngest members of the working population are relatively low in Japan. Yet, in contrast to what is commonly expected, the men/women differences are not higher in Japan. We note that the distribution of PSAs in Japan is heterogeneous. The country has undergone common global influences in terms of the relationship to the body and health and in terms of modern sport and fun wave.

Two important remarks regarding our analysis should be noted: one is that walking is a difficult activity to use in comparisons due to its subjective definition–with the added complication that in our case, walking strongly impacted the overall results. Second, the scope of the comparison (ages 18 to 70) is limited, as it excludes the youngest and oldest, which may skew the results by reducing the importance of recreational activities for young people and thus impact the rate of practice as well as the number of activities practised. To build on these results, a further study could examine the relationship between social characteristics and PSAs, going into greater detail to take into account the modalities of sports practice. This would make it possible to better understand tastes in PSAs specific to Japanese society today, i.e. to gain insight into the Japanese representation of PSAs [66]. For example, even so-called individual sports may be practised collectively in Japan, and the meaning attributed to a practice may differ. For future comparative analyses, in accordance with the analysis of differences in sports participation questioning, four salient explanatory features relating to individuals and organisations could be taken into account to gain more insight into the sports landscape in Japan: 1) the strong cultural context, including the different value system, 2) the organisation of sport at school and university, 3) the employment system, 4) the relationship to the body and the health.
